# White matter abnormalities in adults with bipolar disorder type-II and unipolar depression

**DOI:** 10.1038/s41598-021-87069-2

**Published:** 2021-04-06

**Authors:** Anna Manelis, Adriane Soehner, Yaroslav O. Halchenko, Skye Satz, Rachel Ragozzino, Mora Lucero, Holly A. Swartz, Mary L. Phillips, Amelia Versace

**Affiliations:** 1 Department of Psychiatry, Western Psychiatric Institute and Clinic, University of Pittsburgh Medical Center, University of Pittsburgh, 230 McKee Place, Room 226, Pittsburgh, PA 15213 USA; 2 Department of Psychological and Brain Sciences, Dartmouth College, Hanover, NH USA

**Keywords:** Bipolar disorder, Depression

## Abstract

Discerning distinct neurobiological characteristics of related mood disorders such as bipolar disorder type-II (BD-II) and unipolar depression (UD) is challenging due to overlapping symptoms and patterns of disruption in brain regions. More than 60% of individuals with UD experience subthreshold hypomanic symptoms such as elevated mood, irritability, and increased activity. Previous studies linked bipolar disorder to widespread white matter abnormalities. However, no published work has compared white matter microstructure in individuals with BD-II vs. UD vs. healthy controls (HC), or examined the relationship between spectrum (dimensional) measures of hypomania and white matter microstructure across those individuals. This study aimed to examine fractional anisotropy (FA), radial diffusivity (RD), axial diffusivity (AD), and mean diffusivity (MD) across BD-II, UD, and HC groups in the white matter tracts identified by the XTRACT tool in FSL. Individuals with BD-II (n = 18), UD (n = 23), and HC (n = 24) underwent Diffusion Weighted Imaging. The categorical approach revealed decreased FA and increased RD in BD-II and UD vs. HC across multiple tracts. While BD-II had significantly lower FA and higher RD values than UD in the anterior part of the left arcuate fasciculus, UD had significantly lower FA and higher RD values than BD-II in the area of intersections between the right arcuate, inferior fronto-occipital and uncinate fasciculi and forceps minor. The dimensional approach revealed the depression-by-spectrum mania interaction effect on the FA, RD, and AD values in the area of intersection between the right posterior arcuate and middle longitudinal fasciculi. We propose that the white matter microstructure in these tracts reflects a unique pathophysiologic signature and compensatory mechanisms distinguishing BD-II from UD.

## Introduction

Understanding neurobiological characteristics of distinct mood disorders is critically important yet challenging because of high symptom variability within each disorder, and shared symptoms and patterns of disruption in brain regions supporting cognitive and emotional functioning across diagnoses^1,2^. Discriminating unipolar depression (UD) from bipolar disorder type-II (BD-II) is an important diagnostic distinction which drives treatment decision-making. However, significant symptomatic heterogeneity in each of these conditions makes this differential diagnosis—BD-II vs. UD—particularly challenging in clinical practice. In fact, in about 60% of cases, individuals with BD are initially diagnosed with UD^3^. This is likely due to earlier onset of depression than hypomania, higher prevalence of depressive over hypomanic symptoms, the fact that 64.6% of individuals with UD experience subthreshold hypomanic symptoms such as elevated mood, irritability, and increased energy and activity^4^, as well as identical diagnostic criteria for depressive episodes in the context of BD-II and UD. Misdiagnosis or failures to account for subthreshold mood symptoms may result in inappropriate or delayed treatment and concomitant worsening of symptomatic and functional outcomes.

White matter reorganization may contribute to mood dysregulation, inability to control speech, and problems with attention and memory. Both UD and BD are characterized by poor emotion regulation and impaired cognitive functioning^5–8^. As these processes rely on the network of frontal, striatal, limbic, and parietal brain regions, the integrity of the white matter tracts connecting these regions is of significant interest^9–11^. The main indices of white matter integrity are fractional anisotropy (FA), axial diffusivity (AD), radial diffusivity (RD) and mean diffusivity (MD). Lower FA values indicate that the region has either higher complexity of the fiber architecture (i.e., more crossing fibers), or weaker myelination, or axonal lesion secondary to edema/inflammation, or lower axonal density^12^. Lower AD values could be related to axonal damage while higher RD could be related to a reduced level of myelination or disorganized fiber architecture in the brain^13^.

Previous research has shown that various psychiatric disorders including BD and UD are characterized by reduced integrity of white matter microstructure^14,15^. Specifically, BD, relative to HC, was associated with lower FA in multiple regions including the uncinate, inferior fronto-occipital, inferior and superior longitudinal fasciculi, and cingulum^16–19^. The uncinate fasciculus connects the anterior temporal lobe and amygdala with inferior frontal gyrus and orbitofrontal cortex and is implicated in learning, memory, and emotion regulation^20^. The inferior fronto-occipital fasciculus connects the frontal, insular and occipito-temporal regions in the brain and was implicated in semantic processing^21^ and recognition of facial emotional expressions^22^. The inferior longitudinal fasciculus connects occipital and anterior temporal cortices and plays role in visual cognition including the integration of visual and emotional information^23^. The superior longitudinal fasciculus connects frontal cortices with occipital, parietal, and temporal lobes and is involved in language, memory, attention, and emotion^24^. The cingulum is a large complex tract connecting frontal, medial temporal, limbic, and parietal regions implicated in emotions, memory and executive control^25^. Taken together, reduced white matter integrity in these tracts may contribute to cognitive and emotional dysfunction characterizing mood disorders^26^.

A recent meta-analysis that included 556 individuals with BD and 623 HC found reduced FA in the corpus callosum for BD-I thus, suggesting abnormal reorganization of the fibers in this disorder. However, the small number of BD-II studies prevented the meta-analysis from drawing definitive conclusions about white matter abnormalities in this disorder^27^. The few DWI studies conducted in BD-II have yielded inconsistent results potentially due to using small samples that often included participants across mood states, BD subtypes (BD-I, BD-II, BD-NOS), and age groups (e.g., between 16 and 75 years old). Further, these studies often used less powerful magnets (1.5 T), a small number of DTI directions (e.g., 30), lower b-values (e.g., 1000), and methods of analysis that did not allow for a rigorous correction of geometric distortion, eddy currents and motion. Thus, one study found that FA in the right inferior longitudinal fasciculus was greater in BD-II vs. BD-I^28^, but another study reported that FA in this region was lower in BD-II relative to BD-I and HC^29^. Lower FA values in the inferior and superior longitudinal fasciculi, uncinate fasciculus, inferior fronto-occipital fasciculus^30,31^, interhemispheric and limbic tracts^30^, cingulum and medial prefrontal white matter^28^, and corpus callosum^28,29,31^ have been documented in BD-II relative to HC. Other studies have shown that FA in the uncinate fasciculus was lower in adults with BD-I than in those with BD-II or HC^5,17^. No previous study has compared white matter microstructure among BD-II, UD, and HC or used a dimensional approach^32,33^ to understand how the interaction between depression and spectrum mania symptoms is related to white matter microstructure in these disorders despite that dimensional manifestations of hypomania that may be present over the lifespan in both BD-II and UD^3,34^. Limited attention paid to neurobiological correlates of BD-II could be linked to the difficulty of recruiting and retaining individuals with BD-II in neuroimaging studies as well as the difficulty of distinguishing BD-II from UD. It may also reflect “insufficient understanding of negative consequences of this disorder on individual and public health and insufficient visibility of this disorder to the general public”^35^.

In the current study, we utilized both traditional categorical and mood spectrum approaches to examine white matter abnormalities in mood disorders^34,36–38^. The mood spectrum approach which is consistent with the Research Domain Criteria (RDoC)^33,39^ focuses on a continuous range of psychopathology from sub-threshold to syndromal symptom clusters, traits, and temperamental features to better capture clinical heterogeneity and residual symptoms in mood disorders^34,36–38^. We used state-of-the-art multiband scanning sequences, geometric distortion, eddy currents, and motion correcting procedures, crossing fibers modeling using Bayesian Estimation of Diffusion Parameters Obtained using Sampling Techniques for crossing fibers (*bedpostX*) and XTRACT tool in FSL (FMRIB Software Library^40^). We based the interpretation of our findings on the reconstruction of 42 white matter tracts. This helps overcome the limitations of standard voxel-based approaches (e.g., TBSS or VBM) and assess the extent to which the decreased FA previously reported in individuals with mood disorders^16–19^ is associated with a poor reorganization of the fiber architecture (e.g., miswiring), increased complexity of the fiber collinearity (e.g., crossing, ‘kissing’ and fanning out of the fibers) or myelination deficits. We aimed to (1) characterize BD-II by comparing FA, AD, RD, and MD values in BD-II, UD, and HC; (2) examine the interaction effect between severity of lifetime depression and spectrum mania symptoms on the white matter tract microstructure measures across BD-II and UD; and (3) map findings on the tracts identified by the XTRACT tool. We hypothesized that FA values will be higher in HC vs. BD-II and UD across multiple white matter tracts^14^, and that FA values in fronto-temporal and occipito-temporal regions would be lower in BD-II than in UD^19^.

## Methods

### Participants

The study was approved by the University of Pittsburgh and Carnegie Mellon University (CMU) Institutional Review Boards. All experiments were performed in accordance with relevant guidelines and regulations. Participants were recruited from the community, universities, and counseling and medical centers using advertisements, referrals, and fliers. Written informed consent was obtained from all participants. Participants were right-handed, fluent in English, and matched on age, sex, and IQ. Right-handed participants had no more than two “left-” or “mixed-handed” responses per Annett’s criteria^41^. HC had no personal or first-degree family history of DSM-5 psychiatric disorders. Symptomatic individuals met DSM-5 criteria either for major depressive or unipolar depression (UD) or bipolar type-II (BD-II) disorders and were depressed at scan (Hamilton Rating Scale for Depression (HRSD-25; scores > 14)^42^. The DWI data were collected from 67 participants meeting the above criteria. Motion, b0 and phase encoding direction outliers as well as the presence of scanning artifacts was investigated using visual examination and the eddy quality control tools ‘quad’ (QUality Assessment for DMRI) and ‘squad’ (Study-wise QUality Assessment for DMRI) in FSL^43^. One HC and one individual with UD were removed from the data analysis due to having more than 1% of total outliers, b-value outliers and/or phase encoding direction outliers as well as the poor data quality based on visual examination. The final dataset consisted of 65 participants: 18 with BD-II, 23 with UD, and 24 HC.

### Clinical assessment

Diagnoses were made by a trained clinician and confirmed by a psychiatrist according to DSM-5 criteria using MINI7.0 (Mini International Neuropsychiatric Interview)^44,45^. Exclusion criteria included a history of head injury, metal in the body, pregnancy, claustrophobia, neurodevelopmental disorders, systemic medical illness, premorbid IQ < 85 per the National Adult Reading Test (NART)^46^, current alcohol/drug abuse, the Young Mania Rating Scale (YMRS)^47^ scores > 10 at scan, and meeting criteria for any psychotic-spectrum disorder. We collected information about age at illness onset (onset of depression for UD and onset of depression and hypomania for BD-II), illness duration, number of mood episodes, and psychotropic medications. Past-week depression symptoms were assessed using the HRSD25^42^. Past-week mania symptoms were assessed using the YMRS^47^. Lifetime depression and lifetime spectrum mania symptomatology was assessed using the mood spectrum self-report questionnaire (MOODS-SR)^37^. Higher scores on these questionnaires indicated more severe symptomatology. A total psychotropic medication load was calculated for each participant, with a greater number of medications and higher dosage corresponding to a greater medication load^48,49^. Table 1 reports means, standard errors and group statistics for participants’ demographic and clinical characteristics including medications.

**Table 1:** Demographic and clinical characteristics.

	BD-II N = 18	UD N = 23	HC N = 24	ANOVA/chi-square BD-II vs. UD. vs. HC	t-test/chi-square BD-II vs. UD
Sex (number females)	13	18	16	χ^2^ = 0.79, p = 0.67	na
Age (years)	24.65 (0.98)	25.28 (1.48)	25.89 (1.44)	F(2,62) = 0.19, p = 0.83	t(39) = **− **0.33, p = 0.74
IQ (NART)	109.41 (1.37	112.3 (1.3)	108.9 (1.4)	F(2,62) = 2.07, p = 0.14	t(39) = **− **1.57, p = 0.12
Current depressive episode duration (in weeks)	14.94 (4.74)	14.48 (4.28)	na	na	t(39) = 0.07, p = 0.94
Number of lifetime mood episodes	8.44 (1.44)	6.04 (0.95)	na	na	t(39) = 1.44, p = 0.16
Number of lifetime episodes of depression	5.72 (1.5)	6.04 (0.95)	na	na	t(39) = **− **0.19, p = 0.85
Number of lifetime episodes of hypomania	2.72 (0.4)	na	na	na	na
Depression onset (years of age)	17.06 (0.8)	19.04 (1.6)	na	na	t(39) = **− **1.04, p = 0.3
Hypo/mania onset (years of age)	21.7 (0.9)	na	na	na	na
Illness duration (years)	7.76 (0.81)	6.23 (0.92)	na	na	t(39) = 1.21, p = 0.23
Past-week depression severity (HRSD-25)	27.78 (1.51)	21.52 (1.08)	1.25 (0.27)	F(2,62) = 196.86, p < 0.001	t(39) = 3.46, p < 0.001
Past-week hypo/mania severity (YMRS)	3.39 (0.6)	1.83 (0.33)	0.38 (0.16)	F(2,62) = 16.23, p < 0.001	t(39) = 2.41, p = 0.02
Lifetime depression (MOODS-SR)	22.33 (0.4)	20.26 (0.6)	1 (0.29)	F(2,62) = 680.79, p < 0.001	t(39) = 2.69, p = 0.01
Lifetime hypo/mania (MOODS-SR)	18.33 (0.82)	9.17 (1.24)	4.38 (0.8)	F(2,62) = 47.23, p < 0.001	t(39) = 5.81, p < 0.001
Mean total medication load	1.72 (0.25)	1.04 (0.26)	na	na	t(39) = 1.82, p = 0.08
Mean number of psychotropic medications	1.39 (0.2)	0.7 (0.17)	na	na	t(39) = 2.64, p = 0.01
Number of participants taking Antidepressants	12	11	na	na	χ^2^ = 1.5, p = 0.23
Number of participants taking Mood stabilizers	5	0	na	na	χ^2^ = 7.3, p = 0.007
Number of participants taking Antipsychotics	0	0	na	na	na
Number of participants taking Benzodiazepines	5	2	na	na	χ^2^ = 2.6, p = 0.1
Number of participants taking Stimulants	1	0	na	na	χ^2^ = 1.3, p = 0.25
Number of participants taking 1 psychotropic medication	6	6	na	na	χ^2^ = 0.3, p = 0.6
Number of participants taking 2 psychotropic medication	8	5	na	na	χ^2^ = 2.4, p = 0.1
Number of participants taking 3 psychotropic medication	1	0	na	na	χ^2^ = 1.3, p = 0.25

The table reports the mean and standard error of mean (SE) in parenthesis.

### Neuroimaging data acquisition

The neuroimaging data were collected at the Scientific Imaging and Brain Research Center at Carnegie Mellon University using a Siemens Verio 3 T scanner with a 32-channel head coil. The DWI data were acquired using a multi-band sequence (factor = 4, TR = 3033 ms, resolution = 2 × 2 × 2mm, b = 2000s/mm^2^, 150 directions, 16 B0 images, 68 slices collected parallel to the AC-PC plane, FOV = 220, TE = 124.6 ms, flip angle = 90°). We collected one image in the AP (anterior-to-posterior) direction and the other one in the PA (posterior-to-anterior) direction.

### Data analyses

#### Clinical data analysis

The demographic and clinical characteristics were compared among groups using a one-way ANOVA or chi-square test, as appropriate. BD-II and UD were compared using a t-test. All analyses were conducted in R (https://www.r-project.org/).

#### DWI data analysis

##### Preprocessing and subject-level analyses

The DWI DICOM images were converted to BIDS dataset using *heudiconv*^50^ and *dcm2niix*^51^. They were then preprocessed using FSL 6.0.3 (installed systemwide on the workstation with GNU/Linux Debian 10 operating system). We used *topup* and *eddy_openmp* (with *–cnr_maps–repol–mb* = *4* options) to correct for eddy current-induced distortions and subject motion, and to identify outliers^52,53^. After correcting the images, a diffusion tensor at each voxel was modeled for each subject using the *dtifit* tool in FSL. Participants’ FA, AD, RD, and MD values were registered to the MNI space template using nonlinear registration that aligned all FA images to a 1 × 1x1mm standard space. Crossing fibers were modeled using *bedpostX*^54^ using the ball-and-stick model with a range of diffusivities^55^ and 3 fibers per voxel. The output of *bedpostX* (the crossing fibers fitted data) was used as an input to the GPU version of the XTRACT (cross-species tractography) tool^56^ to automatically extract the set of 42 tracts in each subject. In addition to using the crossing fibers fitted data from *bedpostX, *XTRACT uses diffusion to standard space registration warp fields to perform probabilistic tractography (using *probtrackx2*) in the subject's native space. The normalized tract density for each tract was stored in the MNI standard space.

##### Group-level analysis

Both the categorical and dimensional group-level analyses were conducted using the *randomise* tool^57^ for nonparametric permutation inference with 5000 permutations. The results were corrected for multiple comparisons using Threshold-Free Cluster Enhancement (TFCE) correction^58^ with FWE-corrected p-values < 0.05 in the mean FA mask thresholded at 0.3 to exclude grey matter voxels and minimize partial volume effect in the mask. In all group-level analyses, participants’ age, sex, and IQ were used as covariates of no interest. The tracts identified using the XTRACT tool were used to understand the tractographic composition of the significant clusters.

The categorical group-level analysis used the F-test to identify the clusters of voxels where FA values were different among BD-II, UD, and HC. We then extracted FA, AD, RD, and MD values from the identified clusters and conducted the follow-up analyses in R. Specifically, we ran an ANCOVA (analysis of covariance) analysis with Group as a predictor and age, sex, and IQ as covariates on each DWI measure in each cluster with p-values that were FDR-corrected across all DWI measures and clusters. For those ANCOVAs that showed significant FDR-corrected effect of Group, we computed between-group contrasts (BD vs. HC, UD vs. HC, BD vs. UD) using the *psycho* version 0.4.91 package in R^59^ with p-values corrected across all contrasts, DWI measures, and clusters using FDR. The dimensional group-level analysis was specifically focused on the interaction effect of lifetime depression and spectrum mania symptoms on FA values across individuals with BD and UD (n = 41). The interaction term was of interest because the effect of lifetime depression severity on white matter microstructure could be moderated by severity of the spectrum mania symptoms with greater abnormality observed in individuals with both high depression and high spectrum mania MOODS-SR scores. The FA, AD, RD, and MD values were then extracted from the statistically significant clusters and used in the regression analyses that modeled the DWI measures from the depression-by-mania interaction. As in the first analysis, all p-values were FDR-corrected.

Exploratory analyses examined the effect of the total medication load, illness onset, illness duration, and the total number of mood episodes on each significant result in individuals with BD-II and UD. The brain data were visualized using the “mricron’ software^60^. The interactions were visualized using the *visreg* R package^61^. The other plots were created using the *ggpubr*^62^ and *ggplot2*^63^ packages in R.

## Results

### Clinical

BD-II, UD, and HC groups did not differ from each other in age, IQ, or sex composition (Table 1). Individuals with BD-II and UD endorsed significantly greater past-week and lifetime severity of manic and depressive symptoms than HC (p < 0.05). Individuals with BD-II had more severe symptoms of depression and hypomania at the time of scan and lifetime and took more psychotropic medications (mostly due to taking mood stabilizers) than those with UD (p < 0.05).

### DWI

#### The categorical approach: BD-II vs. UD vs. HC

**Figure 1:**
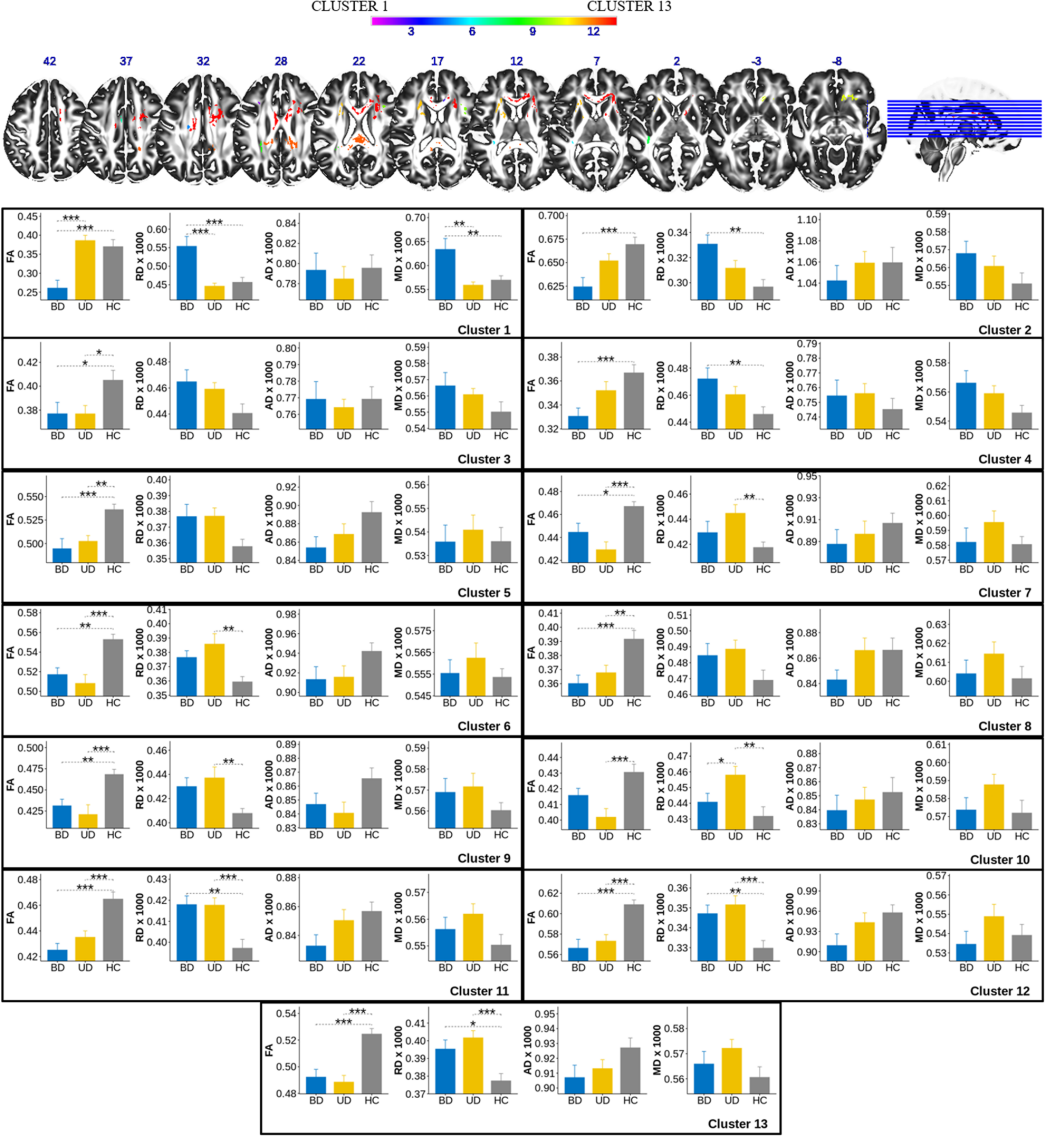
FA, RD, AD, and MD values in the clusters of voxels with the significant Group effect on FA. *q < 0.05, **q < 0.01, ***q < 0.001.

There was a significant main effect of group on FA values in 13 statistically significant clusters of voxels that ranged in size from 14 to 5752 voxels (Table 2, Fig. 1). Clusters overlapped with a total of 24 tracts identified by the XTRACT tool (Table 3). The most representative tracts with over 100 voxels across all significant clusters included bilateral *association fibers* arcuate fasciculus (af_l, af_r), frontal aslant (fa_l, fa_r), inferior fronto-occipital fasciculus (ifo_l, ifo_r), middle longitudinal fasciculus (mdlf_l, mdlf_r), bilateral *projection fibers* anterior (atr_l, atr_r) and superior (str_l, str_r) thalamic radiation, and *commissural fibers* forceps minor (fmi). The main group effect was also observed in the bilateral optic radiation (or_l, or_r), right uncinate fasciculus (uf_r), and dorsal cingulum (cbd_r), which had more than 10 but less than 50 voxels across significant clusters. The least representative tracts that had 10 or less significant voxels across all significant clusters included the left dorsal cingulum (cbd_l), bilateral corticospinal tract (cst_l, cst_r), forceps major (fma), left inferior longitudinal fasciculus (ilf_l), branch 3 of the right superior longitudinal fasciculus (slf3_r), and left uncinate fasciculus (uf_l). Table 3 provides details about the cluster extension, number of participants with common voxels in each tract, as well as the number of voxels showing the main effect of group in each tract. We would like to note that several tracts could cross and intersect within a cluster, therefore, some voxels in the clusters belonged to multiple (often more than two) tracts.

**Table 2:** Comparison of FA, AD, RD, and MD in bipolar disorder type-II, unipolar depression, and healthy controls.

Cluster	Measure	F	p-uncor F-test	q F-test	df	t	p-uncor t-test	q t-test	Summary
**Cluster 1** N = 14 vicinity of af_l	FA	13.69	0.000013	0.000169	59	**− 4.307**	**0.000063**	**0.000349**	**BD < HC**
						− 0.939	0.351637	0.40994	HC = UD
						**− 5.081**	**0.000004**	**0.000072**	**BD < UD**
	MD	8.82	0.000446	0.001933	59	**3.276**	**0.001766**	**0.004102**	**BD > HC**
						0.602	0.54927	0.58158	HC = UD
						**3.759**	**0.000394**	**0.001182**	**BD > UD**
	RD	12	0.000042	0.000437	59	**4.03**	**0.000162**	**0.000648**	**BD > HC**
						0.572	0.569691	0.59446	HC = UD
						**4.465**	**0.000037**	**0.000266**	**BD > UD**
**Cluster 2** N = 18 atr_r, cbd_r, fmi	FA	7.3	0.001465	0.004761	59	**− 3.964**	**0.000202**	**0.000727**	**BD < HC**
						2.03	0.046919	0.071876	HC = UD
						− 1.95	0.0559	0.082407	BD = UD
	RD	6.82	0.002167	0.006549	59	**3.784**	**0.000363**	**0.001136**	**BD > HC**
						− 2.094	0.040545	0.066346	HC = UD
						1.714	0.091718	0.124598	BD = UD
**Cluster 3** N = 26 af_l, str_l	FA	4.4	0.016496	0.037295	59	**− 2.602**	**0.011703**	**0.021606**	**BD < HC**
						**2.806**	**0.006783**	**0.012852**	**HC > UD**
						0.108	0.914594	0.914594	BD = UD
**Cluster 4** N = 36 af_l, cst_l, str_l	FA	6.76	0.002267	0.006549	59	**− 3.887**	**0.00026**	**0.000891**	**BD < HC**
						1.809	0.075481	0.104512	HC = UD
						− 2.083	0.041615	0.066584	BD = UD
	RD	4.15	0.020599	0.044631	59	**3.015**	**0.003787**	**0.00802**	**BD > HC**
						− 2.036	0.046282	0.071876	HC = UD
						1.02	0.311811	0.375493	BD = UD
**Cluster 5** N = 62 af_l, ifo_l, mdlf_l	FA	9.8	0.000211	0.001372	59	**− 4.128**	**0.000117**	**0.000526**	**BD < HC**
						**3.326**	**0.001519**	**0.003646**	**HC > UD**
						− 0.889	0.377415	0.426012	BD = UD
**Cluster 6** N = 90 vicinity of cbd_l	FA	11.07	0.000083	0.000654	59	**− 3.422**	**0.001135**	**0.002818**	**BD < HC**
						**4.534**	**0.000029**	**0.000248**	**HC > UD**
						0.936	0.353004	0.40994	BD = UD
	RD	6.47	0.002887	0.007901	59	2.106	0.039448	0.066052	BD = HC
						**− 3.725**	**0.000439**	**0.001232**	**HC < UD**
						− 1.456	0.150623	0.200831	BD = UD
**Cluster 7** N = 115 af_l, mdlf_l	FA	10.99	0.000088	0.000654	59	**− 2.829**	**0.006364**	**0.012384**	**BD < HC**
						**4.952**	**0.000006**	**0.000086**	**HC > UD**
						1.907	0.061395	0.086675	BD = UD
	RD	4.8	0.011736	0.02774	59	1.397	0.167509	0.219285	BD = HC
						**− 3.505**	**0.000878**	**0.002258**	**HC < UD**
						− 1.939	0.057227	0.082407	BD = UD
**Cluster 8** N = 214 af_l, ifo_l, ilf_l, mdlf_l, or_l	FA	9.28	0.000313	0.001628	59	**− 4.273**	**0.000071**	**0.000365**	**BD < HC**
						**3.721**	**0.000445**	**0.001232**	**HC > UD**
						− 0.659	0.512459	0.550702	BD = UD
**Cluster 9** N = 270 af_r, fa_r	FA	8.44	0.000596	0.002201	59	**− 3.036**	**0.003565**	**0.007778**	**BD < HC**
						**4.054**	**0.000149**	**0.000631**	**HC > UD**
						0.86	0.393062	0.435392	BD = UD
	RD	4.94	0.010376	0.025693	59	2.167	0.034251	0.058716	BD = HC
						**− 3.184**	**0.002319**	**0.005218**	**HC < UD**
						− 0.887	0.378677	0.426012	BD = UD
**Cluster 10** N = 428 af_r, fmi, ifo_r, uf_r	FA	8.47	0.000583	0.002201	59	− 2.172	0.033883	0.058716	BD = HC
						**4.314**	**0.000062**	**0.000349**	**HC > UD**
						1.946	0.056395	0.082407	BD = UD
	RD	5.94	0.004473	0.01163	59	1.174	0.245021	0.3095	BD = HC
						**− 3.837**	**0.000306**	**0.001001**	**HC < UD**
						**− 2.469**	**0.016459**	**0.029626**	**BD < UD**
**Cluster 11** N = 1013 af_l, atr_l, fa_l, fmi, ifo_l, str_l, uf_l	FA	15.8	0.000003	0.000078	59	**− 5.409**	**0.000001**	**0.000036**	**BD < HC**
						**4.515**	**0.000031**	**0.000248**	**HC > UD**
						− 1.018	0.312911	0.375493	BD = UD
	RD	9.51	0.000263	0.00152	59	**3.603**	**0.000646**	**0.001723**	**BD > HC**
						**− 3.981**	**0.000191**	**0.000724**	**HC < UD**
						− 0.238	0.812573	0.824018	BD = UD
**Cluster 12** N = 1945 af_l, af_r, cbd_r, fma, mdlf_l, mdlf_r, or_r	FA	15.05	0.000005	0.000087	59	**− 5.15**	**0.000003**	**0.000072**	**BD < HC**
						**4.807**	**0.000011**	**0.000113**	**HC > UD**
						− 0.491	0.624988	0.642845	BD = UD
	RD	8.36	0.000635	0.002201	59	**2.97**	**0.004307**	**0.00886**	**BD > HC**
						**− 4.164**	**0.000103**	**0.000494**	**HC < UD**
						− 1.028	0.30799	0.375493	BD = UD
**Cluster 13** N = 5752 af_l, af_r, atr_l, atr_r, cbd_l, cbd_r, cst_r, fa_r, fmi, ifo_r, slf3_r, str_l, str_r	FA	17.87	0.000001	0.000052	59	**− 4.8**	**0.000011**	**0.000113**	**BD < HC**
						**5.793**	** < 0.00001**	** < 0.00001**	**HC > UD**
						0.778	0.439516	0.479472	BD = UD
	RD	9	0.000388	0.001834	59	**2.903**	**0.005198**	**0.010396**	**BD > HC**
						**− 4.381**	**0.000049**	**0.000321**	**HC < UD**
						− 1.298	0.199224	0.256145	BD = UD

Values in bold correspond to the entries which passed FDR q < 0.05 threshold.

**Table 3:** The overview of the tracts identified by the XTRACT tool.

	Tract name	Tract abbreviation	N subjects with common voxels in the tract (%)	Tract size (number of 1 mm^3^ voxels)	Cluster numbers with which each tract overlaps	Total number of voxels in each region of the overlap between the tract and the cluster mask	Tracts with significant depression by mania interaction effect in BD-II and UD
	Anterior commissure	ac	60 (92%)	216,515			
Left	Arcuate fasciculus	af_l	65 (100%)	1,147,010	1, 3, 4, 5, 7, 8, 11, 12, 13	1232	
Right	Arcuate fasciculus	af_r	65 (100%)	1,122,237	9, 10, 12, 13	2305	105
Left	Acoustic radiation	ar_l	65 (100%)	67,235			
Right	Acoustic radiation	ar_r	64 (98%)	53,455			
Left	Anterior thalamic radiation	atr_l	65 (100%)	620,525	11, 13	474	
Right	Anterior thalamic radiation	atr_r	65 (100%)	677,637	2, 13	767	
Left	Cingulum subsection: Dorsal	cbd_l	65 (100%)	886,079	6, 13	6	
Right	Cingulum subsection: Dorsal	cbd_r	65 (100%)	860,719	2, 12, 13	34	
Left	Cingulum subsection: Peri-genual	cbp_l	62 (95%)	65,732			
Right	Cingulum subsection: Peri-genual	cbp_r	64 (98%)	56,111			
Left	Cingulum subsection: Temporal	cbt_l	65 (100%)	180,272			
Right	Cingulum subsection: Temporal	cbt_r	65 (100%)	252,177			
Left	Corticospinal tract	cst_l	65 (100%)	305,108	4	5	
Right	Corticospinal tract	cst_r	65 (100%)	329,923	13	2	
Left	Frontal aslant	fa_l	65 (100%)	260,968	11	152	
Right	Frontal aslant	fa_r	65 (100%)	259,002	9, 13	567	
	Forceps major	fma	65 (100%)	546,125	12	10	
	Forceps minor	fmi	65 (100%)	514,563	2, 10, 11, 13	451	
Left	Fornix	fx_l	58 (89%)	61,327			
Right	Fornix	fx_r	55 (85%)	44,647			
Left	Inferior fronto-occipital fasciculus	ifo_l	65 (100%)	930,520	5, 8, 11	87	
Right	Inferior fronto-occipital fasciculus	ifo_r	65 (100%)	953,446	10, 13	225	
Left	Inferior longitudinal fasciculus	ilf_l	65 (100%)	807,135	8	10	
Right	Inferior longitudinal fasciculus	ilf_r	65 (100%)	819,115			
	Middle cerebellar peduncle	mcp	63 (97%)	349,314			
Left	Middle longitudinal fasciculus	mdlf_l	65 (100%)	787,657	5, 7, 8, 12	178	
Right	Middle longitudinal fasciculus	mdlf_r	65 (100%)	851,947	12	80	55
Left	Optic radiation	or_l	65 (100%)	504,901	8	45	
Right	Optic radiation	or_r	65 (100%)	499,767	12	18	
Left	Superior Longitudinal Fasciculus: branch 1	slf1_l	65 (100%)	555,349			
Right	Superior Longitudinal Fasciculus: branch 1	slf1_r	65 (100%)	587,507			
Left	Superior Longitudinal Fasciculus: branch 2	slf2_l	62 (95%)	275,177			
Right	Superior Longitudinal Fasciculus: branch 2	slf2_r	62 (95%)	320,801			
Left	Superior Longitudinal Fasciculus: branch 3	slf3_l	65 (100%)	538,338			
Right	Superior Longitudinal Fasciculus: branch 3	slf3_r	65 (100%)	572,540	13	1	
Left	Superior thalamic radiation	str_l	65 (100%)	309,191	3, 4, 11, 13	183	
Right	Superior thalamic radiation	str_r	65 (100%)	305,351	13	641	
Left	Uncinate fasciculus	uf_l	65 (100%)	392,942	11	3	
Right	Uncinate fasciculus	uf_r	65 (100%)	376,991	10	34	
Left	Vertical occipital fasciculus	vof_l	65 (100%)	310,030			
Right	Vertical occipital fasciculus	vof_r	65 (100%)	277,409			

The follow-up analyses showed the group effect on RD in 10 out of 13 significant clusters, one cluster with the group effect on MD, and no group effect upon AD. The follow-up analysis of the contrasts between the groups (BD-II vs. HC, HC vs. UD, BD-II vs. UD) revealed that whenever there was a significant difference between the groups, FA values were always lower in BD-II and UD vs. HC, while RD values were always higher in BD-II and UD vs. HC. There were two clusters that showed significant differences between BD-II and UD. In Cluster 1 (af_l), BD-II showed significantly lower FA, but significantly higher MD and RD values than both UD and HC. In Cluster 10 (af_r, fmi, ifo_r, and uf_r), BD-II and UD did not differ in their FA values, but UD had significantly higher RD values than BD-II and HC.

#### The dimensional approach: the effect of lifetime depression and spectrum hypomania in BD-II and UD

**Figure 2:**
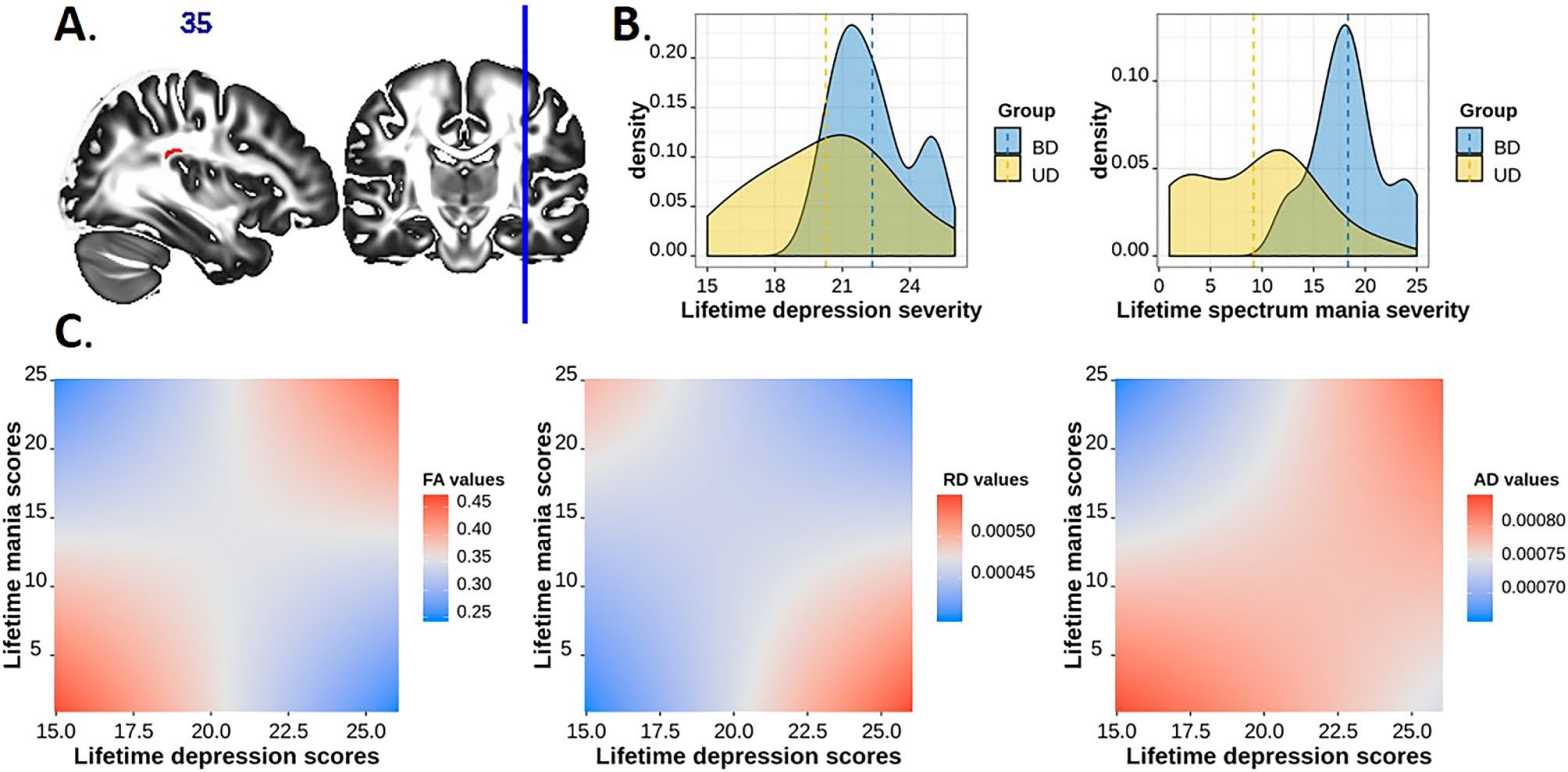
The effect of lifetime depression by lifetime spectrum mania interaction on FA, RD, and AD values in the cluster spanning the right middle longitudinal fasciculus and the right arcuate fasciculus. (**A**) The region of significant depression-by-mania effect on FA values in BD-II and UD. (**B**) The density plots of lifetime depression severity and lifetime spectrum mania scores (per MOODS-SR) in BD-II and UD participants. (**C**) The illustration of significant depression-by-mania interaction effect on FA, RD, and AD values in BD-II and UD. FA in this cluster was greatest and RD values were lowest at low levels of both lifetime depression and mania and high levels of both lifetime depression and mania; AD values were lowest at high levels of mania and low depression.

A significant depression-by-hypomania interaction effect on FA values was observed in one cluster of voxels in the area of intersection between the posterior portion of the right arcuate fasciculus and the right middle longitudinal fasciculus (Fig. 2A). There was a U-shaped relationship between FA (ranged between 0.25 and 0.45) and spectrum depression and mania symptom severity (Fig. 2C). The highest FA values were observed in mood disordered individuals with lowest lifetime severity of depression and spectrum mania symptoms and those with highest lifetime severity of depression and spectrum mania symptoms. These FA values were slightly higher than those in HC (FA = 0.388 ± 0.01). Lower FA values were observed in mood disordered individuals who had high severity of depression but low spectrum mania, and those with low severity of depression but high spectrum mania. The FA values in these individuals were lower than those in HC.

The significant interaction effect on FA was related to the significant depression-by-mania interaction effect on RD (coeff = -0.000001, t(34) = − 3.97, p = 0.00035, q = 0.001), and AD (coeff = 0.000001, t(34) = 2.81, p = 0.008, q = 0.016) in the region described above. The lowest RD values were observed in mood disordered individuals with lowest lifetime severity of depression and spectrum mania symptoms and those with highest lifetime severity of depression and spectrum mania. The lowest AD values were observed in individuals with low lifetime severity of depression but high severity of spectrum mania symptoms.

Given that the majority of individuals with high spectrum mania scores were BD-II, while those with low spectrum mania scores were UD (Fig. 2B), we explored whether the group status was associated with the fiber reorganization in the intersection of the posterior portion of the right arcuate fasciculus and the right middle longitudinal fasciculus described above. We conducted a mixed-effect analysis of normalized densities (a waytotal normalized fiber probability distribution) with the tract (right arcuate fasciculus and right middle longitudinal fasciculus) as a within-subject factor and group (BD-II and UD) as a between-subject factor. There was the main effect of tract showing that the normalized density in the arcuate fasciculus was significantly higher than that in the middle longitudinal fasciculus (F(1,75) = 113.6, p < 0.001, af_r-mdlf_r difference = 0.0068(0.0006), t(39) = 10.7, p < 0.001), but no effect of group or tract-by-group interaction.

#### The effects of the total number of mood episodes, total medication load, age of illness onset, or illness duration in BD-II and UD

There was no statistically significant relationship observed between white matter microstructure measures (i.e., FA and normalized density) in the tracts described above and the total number of mood episodes, total medication load, age of illness onset, or illness duration in BD-II and UD.

## Discussion

In this study, we compared FA values in BD-II, UD, and HC and conducted a depression-by-mania interaction analysis of FA across BD-II and UD. We further used RD, AD and MD values to interpret the statistically significant findings in FA. The automatically segmented white matter tracts^56^ were used to spatially map the clusters of voxels showing either a significant main effect of group or significant depression-by-mania interaction. In line with our first hypothesis, we found that both groups of patients consistently showed decreased FA and increased RD in *association* (bilateral arcuate, inferior fronto-occipital, middle longitudinal fasciculi, and frontal aslant), *projection* (bilateral optic radiation, and bilateral anterior and superior thalamic radiation), *commissural* (forceps minor), and *limbic* (right dorsal cingulum) fibers. Confirming our second hypothesis, the BD-II and UD groups had distinct patterns of white matter alterations in the left arcuate fasciculus and the area of intersection between right arcuate, inferior fronto-occipital, uncinate fasciculi and forceps minor. The combination of different severity levels of lifetime depression and spectrum mania symptoms contributed to the alterations in the white matter microstructure in the area of intersection between the right posterior arcuate and middle longitudinal fasciculi.

Our results are consistent with previous research showing that participants with BD II/NOS, compared to HC, had significant reductions in FA in the major white matter tracts^31^, and that white matter microstructure in the anterior thalamic radiation connecting thalamus to the frontal cortex^64^ and arcuate fasciculus connecting temporal and parietal cortices to the frontal cortex^65^ was associated with depressive symptoms. The frontal aslant tract connects the posterior part of the inferior frontal gyrus with supplementary and pre-supplementary motor areas^66^ and is frequently associated with speech, language, and verbal fluency^67,68^. Our findings of reduced FA, but increased RD, in this tract suggest a potential mechanism underlying well-documented impairments in verbal fluency and information processing in individuals with a history of mood disorders^7,69^.

The findings characterizing the differences between BD-II and UD were of special interest for this study. As we predicted, FA was lower and RD was higher in BD-II than UD and HC in the left arcuate fasciculus that connects frontal, temporal and parietal cortices. Given that this tract was implicated in emotion regulation^70^ including regulation of anger and aggression^71^, reduced integrity of this region may explain more severe mood dysregulation and instability in BD-II vs. UD. An unexpected finding was that UD and BD-II had comparable FA values, but UD had significantly higher RD values than BD-II and HC in the cluster of voxels intersecting the right arcuate, uncinate and inferior fronto-occipital fasciculi, and forceps minor. An increase in RD might be related to decrease in the level of myelination or increase in the axonal diameter or density. The right inferior fronto-occipital fasciculus is involved in non-semantic cognition^72^, uncinate fasciculus in emotional empathy^73^, and arcuate fasciculus in understanding facial emotional expressions^74^. Increased RD in these regions might reflect the difficulty of individuals with UD to understand other people’s emotions and their ability to participate in social and affective communication. Taken together these findings are consistent with the idea that BD-II and UD might have different white matter abnormalities^75^ leading to mood instability in BD-II and deficits in theory of mind in UD^76,77^.

Our dimensional analysis provided the evidence that FA, RD, and AD in the cluster of voxels located at the intersection of the posterior portion of the right arcuate fasciculus and middle longitudinal fasciculus were sensitive to lifetime burden of depression and spectrum mania symptoms across BD-II and UD patients. The role of the arcuate fasciculus in mood disorders was discussed above. The middle longitudinal fasciculus, which extends from the angular to the temporal pole through the superior temporal gyrus^78^, is thought to be implicated in language, memory, and motivation^79^ that is reduced during depression, but increased during hypomania. Interestingly, the lowest FA (and highest RD) were observed in the patients with the most severe depression but least severe spectrum mania and in those with most severe lifetime spectrum mania symptoms but least severe depression symptoms. Their FA values was lower than those in HC. Patients with high depression and high spectrum mania score, and those with low depression and low spectrum mania scores had high FA (but low RD). These patients’ FA was higher than that in HC. These results suggest a possible U-shaped alterations in white matter integrity in the intersection between the right arcuate fasciculus and right middle longitudinal fasciculus. Given that FA for HC was right in the middle, the increase in FA for most symptomatic patients (the majority of who had BD-II) might play a compensatory role by supporting psychosocial functioning through increased motivation. Consistent with previous research^80^, total medication load did not explain white matter microstructure differences in BD-II and UD. However, we have to point out that none of our participants was on lithium, which may increase FA in some white matter tracts^16,80^.

It is thought that a decrease in AD values could be related to axonal damage while an increase in RD values could be related to a reduced level of myelination or miswiring in the brain^13^. In our study, AD values did not depend on the diagnostic group, which is inconsistent with the recent report^81^ that BD, compared to HC, had lower AD in the left posterior thalamic radiation, superior longitudinal fasciculus, inferior longitudinal fasciculus, fronto-occipital fasciculus, and internal capsule. The differences in findings could be explained with the differences in BD sample. While our sample only included BD-II, the sample in^81^ included BD-I, BD-II, and BD-NOS. It is possible that AD abnormalities characterize BD-I but not BD-II. Unlike AD, the RD increases were observed in BD-II and UD relative to HC across multiple clusters of voxels and tracts but were not associated with differences in normalized fiber density (at least in the cluster comprised of the right arcuate and middle longitudinal fasciculus), pinpointing to a possible myelination deficit rather than fiber architecture reorganization. While this interpretation is consistent with the recent proposal of aberrant myelin plasticity in BD^82^, further examination of myelin in these disorders using modern in vivo methods (e.g., using the T1/T2 ratio^83^) is necessary.

### Strengths and limitations

The main strengths of this study include comparing white matter microstructure in BD-II, UD, and HC, using both the categorical and dimensional approaches to psychopathology as well as using state-of-the-art scanning sequences, relatively small voxel size (2 × 2 × 2 mm^3^), and implementing rigorous DWI analysis. We used tractography to interpret findings and map the location of significant effects identified in the whole brain on reconstructed white matter tracts. This has allowed us to assess the extent to which the decreased FA previously reported in individuals with mood spectrum disorders is associated with a reorganization of the fiber architecture (e.g., miswiring), increased complexity of the fiber collinearity, or myelination deficits. The limitations include the cross-sectional design and a relatively small sample size. However, our rigorous methods of analyses that included nonparametric permutation inference and multiple comparisons corrections for the number of voxels and for the number of analyses reduced risk for false positives. Future longitudinal studies should examine the causal relationship between spectrum depression and hypomania symptoms and the changes in white matter microstructure in individuals with BD-II and UD. Specifically, it would be important to understand whether the increases in RD values in mood disordered individuals are associated with the changes in the level of brain myelination in the tracts supporting emotion regulation and cognitive function, or some other reasons.

In summary, we showed that mood disordered individuals may have aberrant white matter integrity in the tracts supporting emotion regulation and cognitive function independently of BD-II and UD diagnosis. We also showed that BD-II and UD could be distinguished based on the patterns of white matter abnormality in bilateral arcuate, right inferior fronto-occipital, and right uncinate fasciculi and forceps minor. The dimensional approach revealed the interaction between lifetime depression and spectrum mania is related to the changes in FA, RD, and AD in the area of intersection between the right arcuate and middle longitudinal fasciculi. We propose that the white matter reorganization in these tracts reflects a unique pathophysiologic signature and compensatory mechanisms distinguishing BD-II from UD. Our study shows that the categorical and dimensional approaches are complementary and that the dimensional approach might constitute an alternative and potentially more physiologically valid strategy to identifying differences within a mood disordered population which, ultimately, could inform more effective treatment (Table S1).

## Supplementary Information

Supplementary Information
