# Supplementary material for: White matter abnormalities in adults with bipolar disorder type-II and unipolar depression
Source: Sci Rep. 2021 Apr 6;11:7541. doi: 10.1038/s41598-021-87069-2 (PMC8024340; doi:10.1038/s41598-021-87069-2)
Supplement: Supplementary file 1 — Supplementary Information [file 41598_2021_87069_MOESM1_ESM.docx]

**Title**: White matter abnormalities in adults with bipolar disorder type-II and unipolar depression

**Authors**: Anna Manelis, Ph.D.^1*^, Adriane Soehner, Ph.D.^1^, Yaroslav O. Halchenko, Ph.D.^2^, Skye Satz, B.S. ^1^, Rachel Ragozzino, M.A. ^1^, Mora Lucero, B.S. ^1^, Holly A. Swartz, MD^1^, Mary L. Phillips, MD, MD (Cantab)^1^, Amelia Versace, MD^1^

**Affiliations**: 1. Department of Psychiatry, Western Psychiatric Institute and Clinic, University of Pittsburgh Medical Center, University of Pittsburgh, Pittsburgh, PA, USA; 2. Department of Psychological and Brain Sciences, Dartmouth College, NH, USA; * corresponding author

**Corresponding author's contact information:** Anna Manelis, Department of Psychiatry, Western Psychiatric Institute and Clinic, 230 McKee Place, Room 226, Pittsburgh, PA 15213, USA.

Email: [anna.manelis@gmail.com](mailto:anna.manelis@gmail.com)

**Running title**: white matter microstructure in mood disorders

**Word count in abstract:** 264 words

**Word count in main text:** 4581

**Number of tables:** 3

**Number of figures:** 2

**Supplemental materials:** 1

# SUPPLEMENTAL MATERIALS

Table S1

FA, RD, AD, and MD values (means and standard errors) in in bipolar disorder type-II (BD-II), unipolar depression (UD), and healthy controls (HC)

|  | **BD-II** | **UD** | **HC** |
| --- | --- | --- | --- |
| **Main effect of Group (categorical analysis across all participants)** | | | |
| **FA Mean Values (SE)** | | | |
| CLUSTER1 | 0.261(0.021) | 0.386(0.014) | 0.37(0.019) |
| CLUSTER2 | 0.624(0.01) | 0.652(0.008) | 0.669(0.008) |
| CLUSTER3 | 0.377(0.01) | 0.377(0.007) | 0.405(0.008) |
| CLUSTER4 | 0.331(0.007) | 0.352(0.007) | 0.367(0.007) |
| CLUSTER5 | 0.495(0.011) | 0.502(0.006) | 0.536(0.006) |
| CLUSTER6 | 0.517(0.007) | 0.508(0.009) | 0.553(0.005) |
| CLUSTER7 | 0.444(0.008) | 0.429(0.007) | 0.467(0.004) |
| CLUSTER8 | 0.36(0.006) | 0.368(0.005) | 0.392(0.006) |
| CLUSTER9 | 0.431(0.008) | 0.421(0.012) | 0.468(0.006) |
| CLUSTER10 | 0.416(0.004) | 0.402(0.005) | 0.431(0.005) |
| CLUSTER11 | 0.425(0.005) | 0.435(0.005) | 0.465(0.006) |
| CLUSTER12 | 0.566(0.009) | 0.573(0.006) | 0.609(0.005) |
| CLUSTER13 | 0.492(0.006) | 0.488(0.005) | 0.524(0.004) |
| **RD Mean Values (SE)** | | | |
| CLUSTER1 | 0.0005538(0.0000265) | 0.0004462(0.0000081) | 0.0004567(0.0000131) |
| CLUSTER2 | 0.0003307(0.0000072) | 0.0003116(0.0000061) | 0.0002967(0.0000058) |
| CLUSTER3 | 0.0004648(0.0000091) | 0.0004591(0.000005) | 0.0004406(0.0000072) |
| CLUSTER4 | 0.0004721(0.0000081) | 0.0004604(0.000006) | 0.0004458(0.0000055) |
| CLUSTER5 | 0.0003767(0.0000078) | 0.000377(0.0000053) | 0.0003577(0.0000046) |
| CLUSTER6 | 0.0003764(0.0000047) | 0.0003858(0.0000073) | 0.0003593(0.0000037) |
| CLUSTER7 | 0.0004293(0.000009) | 0.0004447(0.0000069) | 0.0004175(0.0000043) |
| CLUSTER8 | 0.0004845(0.0000076) | 0.0004886(0.0000056) | 0.000469(0.0000064) |
| CLUSTER9 | 0.0004299(0.0000073) | 0.0004371(0.0000091) | 0.0004077(0.0000041) |
| CLUSTER10 | 0.0004407(0.0000056) | 0.000458(0.0000055) | 0.0004317(0.000006) |
| CLUSTER11 | 0.000418(0.0000041) | 0.0004177(0.0000035) | 0.0003972(0.0000042) |
| CLUSTER12 | 0.0003471(0.0000043) | 0.0003516(0.0000045) | 0.0003297(0.0000038) |
| CLUSTER13 | 0.0003953(0.0000051) | 0.0004017(0.000004) | 0.0003773(0.0000041) |
| **AD Mean Values (SE)** | | | |
| CLUSTER1 | 0.0007934(0.000017) | 0.0007847(0.0000123) | 0.0007955(0.0000131) |
| CLUSTER2 | 0.0010423(0.0000144) | 0.001059(0.0000108) | 0.0010594(0.0000144) |
| CLUSTER3 | 0.0007691(0.0000107) | 0.0007641(0.0000049) | 0.0007692(0.0000075) |
| CLUSTER4 | 0.0007544(0.0000108) | 0.000756(0.0000068) | 0.0007452(0.0000075) |
| CLUSTER5 | 0.0008538(0.000012) | 0.0008685(0.0000114) | 0.0008924(0.0000118) |
| CLUSTER6 | 0.0009132(0.000013) | 0.0009157(0.0000115) | 0.000942(0.0000086) |
| CLUSTER7 | 0.0008875(0.0000133) | 0.0008968(0.0000119) | 0.0009067(0.0000091) |
| CLUSTER8 | 0.0008429(0.0000077) | 0.000866(0.0000097) | 0.0008662(0.0000095) |
| CLUSTER9 | 0.0008468(0.000008) | 0.0008405(0.0000079) | 0.0008654(0.0000077) |
| CLUSTER10 | 0.0008395(0.0000109) | 0.0008471(0.0000089) | 0.0008524(0.0000106) |
| CLUSTER11 | 0.0008327(0.0000077) | 0.0008504(0.0000076) | 0.0008567(0.0000064) |
| CLUSTER12 | 0.0009092(0.0000172) | 0.0009433(0.0000142) | 0.0009578(0.0000117) |
| CLUSTER13 | 0.0009071(0.0000083) | 0.0009131(0.0000061) | 0.0009271(0.0000066) |
| **MD Mean Value (SE)** | | | |
| CLUSTER1 | 0.0006336(0.0000227) | 0.000559(0.0000066) | 0.0005696(0.0000089) |
| CLUSTER2 | 0.0005679(0.0000069) | 0.0005607(0.0000057) | 0.0005509(0.0000061) |
| CLUSTER3 | 0.0005662(0.0000082) | 0.0005608(0.0000037) | 0.0005501(0.0000062) |
| CLUSTER4 | 0.0005662(0.0000085) | 0.0005589(0.0000054) | 0.0005456(0.0000051) |
| CLUSTER5 | 0.0005358(0.0000071) | 0.0005408(0.0000064) | 0.0005359(0.0000059) |
| CLUSTER6 | 0.0005554(0.0000063) | 0.0005624(0.0000069) | 0.0005536(0.0000039) |
| CLUSTER7 | 0.000582(0.0000096) | 0.0005954(0.0000077) | 0.0005806(0.0000053) |
| CLUSTER8 | 0.000604(0.0000072) | 0.0006144(0.0000064) | 0.0006014(0.0000064) |
| CLUSTER9 | 0.0005689(0.0000066) | 0.0005716(0.0000064) | 0.0005603(0.0000037) |
| CLUSTER10 | 0.0005737(0.0000068) | 0.0005877(0.0000058) | 0.000572(0.0000071) |
| CLUSTER11 | 0.0005562(0.0000045) | 0.0005619(0.0000038) | 0.0005503(0.000004) |
| CLUSTER12 | 0.0005345(0.0000067) | 0.0005488(0.0000063) | 0.0005391(0.0000056) |
| CLUSTER13 | 0.0005659(0.000005) | 0.0005721(0.0000035) | 0.0005606(0.0000042) |
| **Depression x Mania Interaction Effects (dimensional analysis across BD-II and UD)** | | | |
| **FA Mean Values (SE)** | | | |
| Right af – mdlf cluster | 0.37(0.008) | 0.371(0.01) | 0.388(0.01) |
| **RD Mean Values (SE)** | | | |
| Right af – mdlf cluster | 0.00045(0.000006) | 0.00045(0.000007) | 0.00044(0.000006) |
| **AD Mean Values (SE)** | | | |
| Right af – mdlf cluster | 0.00077(0.000008) | 0.00077(0.000011) | 0.00078(0.000009) |
| **MD Mean Value (SE)** | | | |
| Right af – mdlf cluster | 0.00055(0.000005) | 0.00056(0.000006) | 0.00055(0.000004) |
